# What do people want to know about NIPT? Content analysis of questions emailed to national NIPT information websites

**DOI:** 10.1002/pd.5011

**Published:** 2017-02-28

**Authors:** Saskia Tamminga, Laura van Dussen, E. J. (Joanne) Verweij, Marjon A. de Boer, Martina C. Cornel, Lidewij Henneman, for the Dutch NIPT Consortium

**Affiliations:** ^1^ Department of Clinical GeneticsVU University Medical CenterAmsterdamthe Netherlands; ^2^ Amsterdam Public Health Research InstituteVU University Medical CenterAmsterdamthe Netherlands; ^3^ ErfocentrumAmersfoortthe Netherlands; ^4^ Department of Obstetrics and GynaecologyLeiden University Medical CenterLeidenthe Netherlands; ^5^ Department of Obstetrics and GynaecologyVU University Medical CenterAmsterdamthe Netherlands

## Abstract

What's already known about this topic?

- Several countries, including the Netherlands, have implemented non‐invasive prenatal testing (NIPT) in their national prenatal screening program
- Access to relevant online health information is important as it increases informed decision making

What does this study add?

- Visitors of national NIPT websites mostly request more information about testing beyond the currently available NIPT: a broader range of disorders (such as monogenic disorders) and increasing target group eligibility (such as low‐risk or twin pregnancies)

© 2017 The Authors. *Prenatal Diagnosis* published by John Wiley & Sons, Ltd.

With non‐invasive prenatal testing (NIPT) cell‐free fetal DNA of placental origin can be detected in maternal plasma. NIPT is an accurate test for detecting fetal trisomy 21, 18 or 13 in both high and low‐risk pregnancies^1^ and, unlike invasive testing, carries no miscarriage risk. As false positives occur, invasive confirmation of a positive test result is needed.

In many countries, NIPT has been commercially introduced without governmental guidance. The Netherlands is one of the first countries to incorporate NIPT into a governmentally supported and healthcare funded prenatal Down syndrome screening program. Prenatal screening is subject to a governmental license under the Dutch Population Screening Act. On 17 December 2013, a license was obtained by the National NIPT Consortium for a nationwide implementation study (TRIDENT study, Trial by Dutch laboratories for Evaluation of Noninvasive prenatal Testing). Since April 2014, NIPT is offered as an alternative option to invasive testing for pregnant women at increased risk of having a child with trisomy 21, 18 or 13 based on the first trimester combined test (cut‐off 1:200) or because of a previous child with these trisomies.^2^ Pre‐ and post‐test counseling is part of the program in order to enhance autonomous decision making.^3^ In the first year of the study, around 3000 tests were performed. Until 2017, NIPT as a first‐tier screening test was not available, which is why many pregnant women choose to pay for commercially offered NIPT in other countries.

Since the start of the TRIDENT study, two websites have been available providing information about NIPT in the Netherlands; the national study website *meerovernipt.nl* and the NIPT Consortium website *niptconsortium.nl*. Besides a page with frequently asked questions, both websites offer visitors the opportunity to ask questions per e‐mail. More than 95% of the Dutch population has access to Internet, and at least half of this group uses the Internet for health‐related issues.^4^ Therefore, the Internet is considered a powerful tool in providing health information. As health information makes individuals engage more in their medical decision making,^5^ we decided to investigate questions asked per e‐mail about NIPT.

The aim of this study was to determine the information needs of visitors of both NIPT websites and to explore possible gaps of knowledge in order to fit health information to their needs by performing content analysis of questions asked. We also analyzed website visitor counts and trends in visits, to evaluate the impact of certain media activities.

*Meerovernipt.nl* is the TRIDENT study website which provides information about NIPT, including information for study participants.^2^ The site is an initiative of the NIPT Consortium and is maintained by the Dutch National Information Center on Heredity (Erfocentrum). The NIPT Consortium is a national collaboration including health professionals, laboratory specialists, patient representatives, ethicists and the national prenatal screening organization. *Niptconsortium.nl* is the portal for members of the Consortium and contains information about Dutch studies on NIPT and (inter)national key publications.

Data were derived from all questions e‐mailed by visitors to both NIPT websites from 17 December 2013 (license approval) until two years later (17 December 2015). All questions were anonymized and assigned an identification number (#). Data were content analyzed and coded independently by two researchers (ST and LvD). In discussion with a third researcher (LH), uncertainties and discrepancies in coding were discussed, and categories were identified and labeled. Data were primarily coded on the topics that people inquired about. Differences and similarities among questions submitted by questioners with different backgrounds were examined. When the background of the questioner (e.g. pregnant women, health professional, student) could not be deduced directly, it was derived from the type of question. For example, ‘I've had NIPT, and I want to know more about […]’ (pregnant woman) or ‘I have a client who is now 9 weeks pregnant and […]’ (health professional). Website visitor counts were obtained from Google Analytics, and time series were obtained for the number of visitors per month.

In the 2‐year period, 233 visitors sent an e‐mail: 174 to the study website *meerovernipt.nl* and 62 to the Consortium website. Three questioners sent the same question to both websites. There was no difference in the background of the visitors between both websites. The majority of questioners were (pregnant) women or their partner/relative (67%), followed by health professionals (16%), students (5%), journalists (5%), researchers (2%) or other (5%), such as providers of other NIPT‐related websites or commercial NIPT providers.

Because (pregnant) women (or their partner/relative) and health professionals accounted for the majority of questioners (83%), for this article, we focused on their 222 questions (Table 1). ‘Is NIPT available for […]’ was the most asked question from both groups (37%), such as a twin pregnancy or genetic disorders other than common trisomies (e.g. chromosomal deletions/duplications or monogenic disorders). More than a quarter of women's questions (28%) were about NIPT in a low‐risk pregnancy. Their questions concerned how, where or when to apply for NIPT without *a priori* high risk, and the costs thereof. Women were also interested in how NIPT works (23%), especially concerning the accuracy of NIPT (44% within this group), while only one question asked by a health professional concerned the accuracy. Health professionals were more interested in how the TRIDENT study works (27%). Ten questions were related to non‐medical fetal sex determination with NIPT.

**Table 1:** Topics that (pregnant) women (or their partner/relative) and health professionals inquire about

Topics	Questions from pregnant women *n = 181* *n* (%)	Questions from health professionals *n = 41* *n* (%)	Total of questions *n = 222* *n* (%)
*Is NIPT available for […]?*	63 (35)	19 (46)	82 (37)
Twin pregnancy/vanishing twin	15	3	18
Genetic disorder other than trisomy 21, 13 or 18^a^	13	2	15
Previous child with (mosaic) trisomy 21, 13 or 18	6	6	12
FCT result^b^	7	3	10
NT ≥ 3.5 mm	5	2	7
Advanced maternal age	5	2	7
Aberrant obstetric history^c^	5	0	5
ICSI pregnancy	4	0	4
Ultrasound abnormalities	3	1	4
*Can NIPT be accessed without (a priori) high risk?*	50 (28)	4 (10)	54 (24)
How/where/when to apply for NIPT	23	1	24
Costs	12	0	12
Possibility out‐of‐pocket payment	8	1	9
Availability (in another country)	7	2	9
*How does NIPT work?*	41 (23)	7 (17)	48 (23)
Accuracy^d^	18	1	19
Reporting test results^e^	7	0	7
Time window for testing	5	3	8
Turnaround time of results	6	0	6
Follow‐up testing	2	0	2
Logistics	1	1	2
Privacy and liability^f^	1	2	3
Aberrant NIPT result	1	0	1
*How does the TRIDENT study work?*	14 (7)	11 (27)	25 (11)
Participation^g^	6	4	10
General information^h^	4	6	10
How/where to apply	4	1	5
*What is the scope of NIPT (non‐medical)?*	13 (7)	0 (0)	13 (6)
Fetal sex^j^	10	0	10
Paternity	3	0	3

NT, nuchal translucency; ICSI, intracytoplasmic sperm injection; FCT, first trimester combined test. Percentages may not add up to 100% due to rounding. Questioners could ask more than one question.

a Monogenic, subchromosomal (deletion and/or duplication) or other (including sex) chromosomal disorders.

b E.g. increased risk at FCT, high risk at FCT in pregnancy from egg donation, risk slightly increased (≥1:200), due to circumstances no FCT performed.

c E.g. previous pregnancy with intra‐uterine fetal demise, previous pregnancy of fetus with cardiac malformation, previous pregnancy with high risk at FCT.

d Accuracy of NIPT in general, accuracy of NIPT compared to FCT or chorionic villus sampling or amniotic puncture, reliability of NIPT when the mother has a high weight or length.

e Specific information included in test result, reporting results for other chromosomes than chromosomes 21, 13 and 18, or reporting no result.

f Liability when NIPT is commercially performed in another country (e.g. Belgium).

g Where to get NIPT in another country, why participation in the TRIDENT study is not allowed, definition of high risk (concerning FCT and/or medical indication).

h Contact details, brochures and questions about informed consent or reimbursement policy.

j Determination of fetal sex with NIPT, discrepancy between fetal sex at ultrasound findings and NIPT conducted in another country.

When in 2013 it was announced that NIPT would be offered in the Netherlands as second‐tier screening test, and after its actual introduction in April 2014, there were high visitor counts on both websites (Fig. 1). This followed widespread media coverage following a year of debate about NIPT. Over time, with some peaks minutes after certain media attention, such as a television program concerning NIPT, the study website *meerovernipt.nl* has seen a significant increase in visits per month, from around 350 in January 2014 to around 1000 in January 2015. This increase underlines that the Internet holds great potential to support health information gathering and decision making. In contrast, visitor counts of the Consortium website declined. Perhaps, visitors of the Consortium website increasingly sought their information on the study website *meerovernipt.nl* or the decline is because health professionals developed more experience with NIPT in their daily clinical practice.

**Figure 1:**
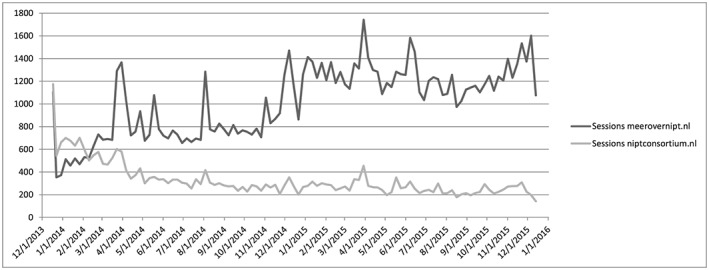
Visitor counts for the study website *meerovernipt.nl* and NIPT Consortium website, from 17 December 2013 until 17 December 2015

As many questions on indications concerned conditions other than the common trisomies, we conclude that there is much interest in the availability for NIPT for conditions other than these aneuploidies. Previous studies have shown that both pregnant women and health professionals favor NIPT for a broader range of disorders.^6^ In the UK, non‐invasive prenatal diagnostics (NIPD) is available for an increasing number of monogenic disorders, such as cystic fibrosis, achondroplasia and craniosynostosis syndromes. In 2014, about one third of prenatal diagnostic tests for monogenic disorders were performed using NIPD in the UK.^7^ The scope of NIPT/D is further expanding, for example for sex chromosome aneuploidies and microdeletion and microduplication syndromes. However, a routine offer is still debated partly because of biological difficulties (such as mosaicism) in sex chromosome aneuploidies and lack of validated studies in microdeletion syndromes.^8^ Furthermore, some of these conditions are less severe and/or have a wide clinical spectrum. In our study, questions about availability of NIPT (such as previous child with a trisomy) could also imply that current information, e.g. online or during pre‐test counseling, is not sufficient. When NIPT/D becomes available for a broader range of disorders, it is important to outline which disorders are tested, in order to enhance well‐informed decision making.

Women asked questions about how and where they could apply for NIPT and what the costs would be, suggesting that they are interested in, and are willing to pay for, NIPT in a low‐risk pregnancy. Many Dutch low‐risk pregnant women (i.e. from the general obstetric population) paid for NIPT commercially offered in other countries, as until 2017 NIPT is not available for low‐risk women. In 2016, the Dutch Ministry of Health decided to expand the license of the TRIDENT study, meaning that NIPT for the common trisomies will be offered to all pregnant women as a first‐tier screening test from presumably Spring 2017.

When looking at questions about how NIPT works, in comparison to only one health professional, many women were interested in the accuracy of NIPT. Earlier research however showed that health professionals consider test accuracy to be the most important feature of a prenatal test.^9^ Possibly, health professionals are already familiar with the test accuracy of NIPT.

Women asked about the non‐medical potential of NIPT, such as gender and paternity. Based on ethical and social considerations, a Dutch NIPT result does not give information on either aspect, as there is no underlying health benefit. Yet, information about fetal sex is sometimes included in commercially offered NIPT. This is a major issue as knowledge about fetal sex might lead to sex selection abortion based on societal values and preferences in certain cultures.^10^

As far as we are aware, this is the first study to analyze NIPT‐related questions of online health information seekers. Data were based on people's need for information using their own words. As access to the websites is nationwide, there is no selection bias for residence. A limitation is lack of demographic data of the questioners, such as age, pregnancy details, educational level or specific type of profession.

In conclusion, Dutch women and health professionals asked most questions concerning the conditions and population eligible for NIPT, suggesting that they are interested in a broader scope of the test than is currently available. This comprises target groups (which pregnancies) on the one side and target disorders (which conditions) on the other side. Online information and pre‐test counseling should pay attention to range and scope of NIPT in order to meet knowledge needs and enhance well‐informed decision making.
